# Habitat and Canopy Position Influence Leaf Traits and Trait-Associations of a Large-Sized Leguminous Herb (*Crotalaria spectabilis*)

**DOI:** 10.3390/plants15030492

**Published:** 2026-02-05

**Authors:** Cheng Wang, Ji-Yuan Liu, Xin-Yue Jin, Meng-Ting Wang, Duo-Qi Zhou, Ye Tao

**Affiliations:** 1 College of Life Sciences, Anqing Normal University, Anqing 246133, China; 2 State Key Laboratory of Ecological Safety and Sustainable Development in Arid Lands, Xinjiang Institute of Ecology and Geography, Chinese Academy of Sciences, Urumqi 830011, China; 3 Xinjiang Key Laboratory of Biodiversity Conservation and Application in Arid Lands, Xinjiang Institute of Ecology and Geography, Chinese Academy of Sciences, Urumqi 830011, China

**Keywords:** functional trait, leguminous herb, habitat heterogeneity, canopy position, trait network, survival strategy

## Abstract

*C. spectabilis* (*Crotalaria spectabilis*), a large leguminous herb species, is widely distributed in tropical and subtropical regions, and it has important ecological and economic values. However, the ecological adaptation of the major leaf functional traits of the species across different habitats and canopy positions remains poorly understood. To address this gap, we sampled leaves from the upper, middle, and lower canopy positions in two common habitats—forest understory and exposed land—and quantified key leaf traits as well as trait–trait relationships to assess differences. The results showed that irradiance and air temperature were significantly lower in the understory than in exposed land, whereas soil moisture and relative humidity were higher, indicating that habitat exerted a stronger influence on leaf traits than canopy position. Canopy position also significantly affected most traits and showed significant interactions with habitat. In exposed land, middle plants exhibited higher individual leaf dry mass (180.049 ± 68.480 mg), larger vein diameter (1.692 ± 0.288 mm), and longer petioles (5.406 ± 0.940 mm). These traits were accompanied by a higher morphology-based leaf dry matter accumulation rate and greater stability of the leaf-trait network, reflecting an adaptive strategy characterized by increased structural investment. In contrast, understory middle leaves were generally longer (13.361 ± 2.714 cm) and wider (7.005 ± 1.464 cm), along with lower photosynthate accumulation rates and weaker trait-network stability, indicating a strategy that enhances light-use efficiency under low-light conditions. In both habitats, leaves from the middle canopy position generally exhibited the highest values for most measured traits. Overall, leaf traits of *C. spectabilis* and their interrelationships showed considerable plasticity in response to external environmental pressures, primarily differences in light availability. However, from a practical production perspective, minimizing shading is recommended to maximize its ecological benefits.

## 1. Introduction

Plant functional traits comprise the biological characteristics that reflect how plants adapt to their environments [1]. Leaves, which present the largest surface area in contact with the environment and constitute the principal photosynthetic organ, have functional traits that reveal plants’ adaptive strategies [2]. The linkage between environmental factors and leaf functions is manifested through leaf traits; specifically, certain functional traits vary to accommodate different environmental conditions [3]. Plant functional traits are closely interrelated and are jointly coordinated and constrained by trade-offs [4], and leaf traits likewise exhibit multidimensional, complex patterns of covariation [5]. Therefore, investigating leaf functional traits helps to elucidate plant–environment relationships, particularly plants’ adaptive strategies to heterogeneous environments.

Under contrasting environmental conditions, the same trait within a given species often exhibits pronounced variation, reflecting phenotypic plasticity [6]. The leaf economics spectrum (LES) quantifies plants’ resource-use capacities and associated trade-off strategies, thereby illustrating patterns of variation and covariation in leaf traits under changing environmental conditions; the two ends of the spectrum represent contrasting plant survival strategies [7]. Plant trait variation is influenced by multiple factors, among which light availability, temperature, and water conditions are typically the most important [8], and together constitute the primary dimensions of habitat heterogeneity. Plants growing under high-light conditions usually develop thicker leaves, as increased palisade tissue is required to protect against damage caused by excessive irradiance [9]. In contrast, under low-light conditions, leaves tend to be larger and thinner, with softer texture, lower leaf mass per area (LMA), and longer petioles [10]. The effects of temperature on leaf functional traits are mainly reflected in leaf development and size: plants in warmer environments generally have larger leaves, whereas low temperatures constrain leaf expansion, resulting in smaller, thicker leaves with higher LMA [11]. With the transition from the dry season to the wet season, evergreen tree species in rainforests exhibit increases in LMA and leaf tissue density, indicating the sensitivity of leaf traits to changes in water availability [8]. Collectively, these findings demonstrate that habitat heterogeneity exerts a strong influence on plant leaf traits.

Light radiation can vary among positions within the canopy; however, within-canopy light gradients are not strictly monotonic and are influenced by canopy structure and optical processes (e.g., multiple scattering and reflection, refraction). Therefore, the magnitude of light differences among canopy positions is context-dependent, reflecting a dynamic and multidimensional interaction between canopy structure and the light environment [12,13]. Plants adapt to surrounding light conditions by adjusting the physiological and morphological traits of their photosynthetic organs in order to maximize leaf function and carbon acquisition [14]. Studies on the anatomical structures of hybrid spruce (*Picea asperata*) [15] have shown that leaves in the upper canopy exhibit the greatest thickness, whereas those in the lower canopy are the thinnest, primarily because upper-canopy leaves receive higher irradiance and thus develop thicker epidermal tissues. At present, research on the effects of canopy position on leaf traits has largely focused on woody plants, particularly trees. In contrast, for herbaceous plants with pronounced vertical stratification, it remains unclear whether their leaves exhibit canopy effects similar to those observed in woody species.

The covariance and trade-off relationship between traits profoundly reflect the overall adaptation of plants to their environments. Allometric scaling relationships capture the intrinsic pairwise associations and covariation patterns among leaf traits [16]. Studies on leaves and petioles of woody species show hypoallometric scaling—scaling exponent (α) < 1—of leaf dry mass, area, and volume against petiole dry mass, indicating that leaf enlargement is constrained by the petiole [17]. In *Ginkgo biloba*, increases in leaf mass and leaf area are asynchronous, and variation in a leaf-shape index (LSI) markedly influences the allometric relationship between leaf mass and area [18]. Across habitats, *Phragmites australis* exhibits slower increases in leaf width than in leaf length; the growth rate of leaf mass approximates that of leaf area, but the exponent for leaf mass versus the LSI (*α* > 1) denotes hyperallometry [16], indicating a relatively consistent scaling pattern. Under contrasting light regimes, trade-offs among traits also shift markedly as irradiance declines, although species at different successional stages adopt distinct adaptive strategies [19]. In addition, plant trait networks (PTNs) provide a network-topological perspective on multivariate trait variation and enable intuitive visualization; they are an emerging tool in plant biology and ecology. Depending on the target set, PTNs can be constructed for leaf traits, root traits, physiological traits, and so on. For instance, along a representative forest transect in eastern China, the diameter, mean path length, and average clustering coefficient of a 34-trait leaf PTN all increase with latitude—that is, the overall connectedness among functional traits is stronger in low-latitude than in high-latitude forests [20]. Consequently, integrating analytical approaches across dimensions can better elucidate how plants reorganize trait relationships under environmental stress.

*C. spectabilis* is a large herbaceous legume widely distributed across tropical and subtropical regions, particularly in Southern and Southeastern Asia. The species has important applications in drug development, as a green manure, and in the petroleum and textile industries, as well as for windbreaks, sand stabilization/erosion control, and ecological restoration [21]. It is also broadly used in agricultural production; for example, intercropping with maize increased nitrogen accumulation in maize straw by 39% [22]. Although habitat heterogeneity has been shown to significantly affect biomass allocation in *C. spectabilis* [14], it remains unclear how leaf functional traits and inter-trait relationships respond to differences in habitat and canopy position. In this study, focusing on populations growing in two habitat types—forest understory and exposed land—we combined field sampling with laboratory analyses to: (1) test for differences in main leaf traits across habitats and canopy positions and evaluate the relative effect sizes of each factor; (2) assess whether allometric scaling relationships among traits differ between habitats and canopy positions; and (3) characterize how overall trait interrelationships vary with habitat and canopy position. Guided by patterns documented for woody plants, we hypothesized that (1) habitat exerts a stronger filter on traits than canopy position; and (2) high-light environments promote stronger trait network connectivity. This study extends well-known light-driven trait patterns by focusing on a large herbaceous legume, explicitly comparing the relative roles of habitat filtering versus within-plant canopy position, and integrating allometry, PCA, and plant trait networks to reveal not only trait shifts but also habitat-dependent changes in trait coordination and network stability. Our findings aim to elucidate the environmental adaptability and self-regulatory capacity of *C. spectabilis* from the perspective of leaf traits and their covariation, and to provide ecological evidence that may inform its further development and utilization.

## 2. Results

### 2.1. Basic Characteristics of Leaf Morphological Traits

In the understory, the coefficients of variation (CVs) for leaf morphological traits ranged from 0.073 to 0.448, whereas in the exposed land they ranged from 0.053 to 0.380 (Table 1). In both habitats, LM exhibited the largest CV, while the LSI showed the smallest. Habitat and canopy position each had highly significant effects on all eight traits, and—except for PD and LA—the main effect of habitat was the largest (Table 2). In addition, habitat × canopy position interactions were highly significant for seven traits (all except PL, PD and LA).

**Figure 1:**
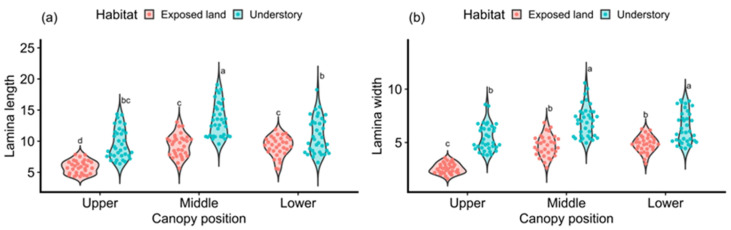

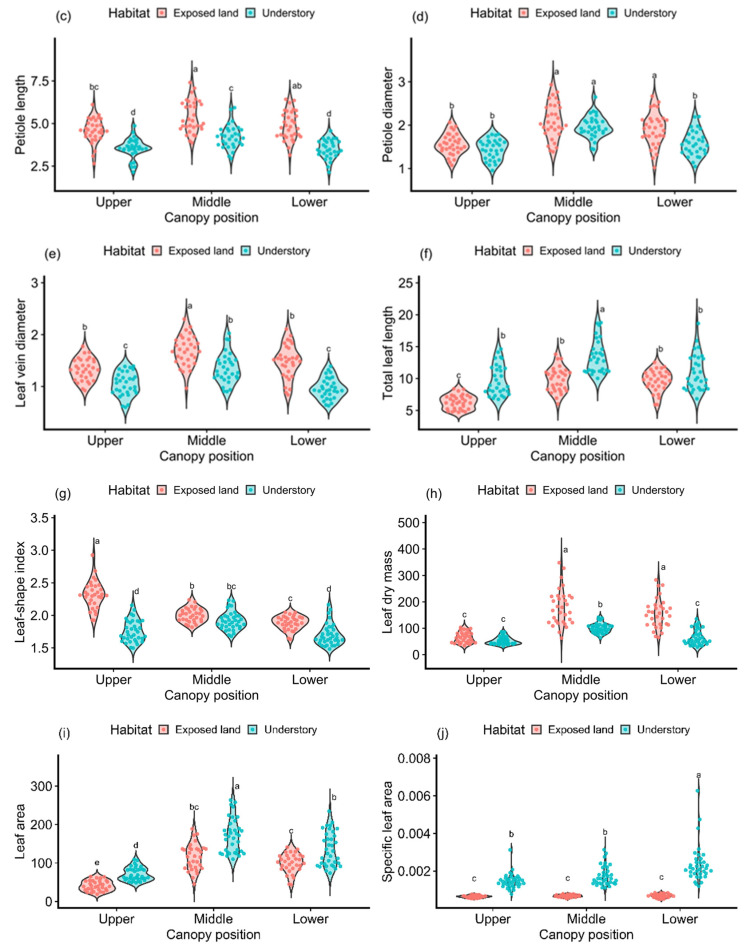
Differences in leaf traits of *Crotalaria spectabilis* in different habitats and canopy positions. (**a**) Lamina length; (**b**) Lamina width; (**c**) Petiole length; (**d**) Petiole diameter; (**e**) Leaf vein diameter; (**f**) Total leaf length; (**g**) Leaf-shape index; (**h**) Individual leaf dry mass; (**i**) Leaf area; (**j**) Specific leaf area (SLA). Different lowercase letters (a–e) indicate statistically significant differences.

Analysis of variance revealed that, across all canopy positions, all measured leaf traits of *C. spectabilis* differed significantly between the two habitats (Figure 1). Specifically, plants growing in exposed land exhibited significantly lower LL, LW, TLL, LA, and SLA than those in the forest understory, whereas the remaining five traits showed the opposite pattern. Within the forest understory, leaves from the middle canopy position exhibited significantly higher values of LL, LW, LVD, PD, TLL, and LA than those from the lower canop. Both LVD and PL reached their highest values in the middle canopy and were significantly greater than those in the upper and lower canopy positions. In exposed land, however, patterns of variation in leaf traits across canopy positions differed from those observed in the understory. PL, PD, LM, and LA exhibited similar trends across the three canopy positions, whereas LL, LW, and TLL were lowest in the upper canopy, with no significant differences between the middle and lower canopy positions. LSI showed a decreasing trend from the upper to the lower canopy, while SLA varied little among canopy positions. Overall, these results indicate that *C. spectabilis* exhibits strong environmental plasticity in leaf traits across both microhabitats and canopy strata.

### 2.2. Correlations Among Leaf Traits and Differences in Allometric (Scaling) Exponents

**Figure 2:**
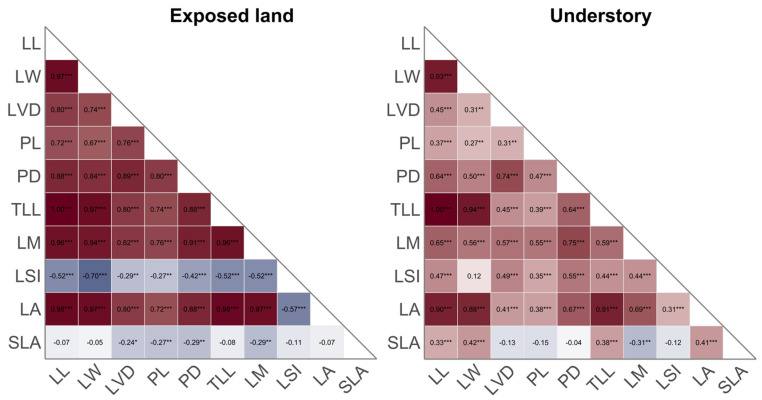
Pearson correlation heatmaps of leaf traits of *Crotalaria spectabilis* in two habitats. The color gradient represents the magnitude of correlation coefficients, where red and blue denote positive and negative correlations, respectively. ***: *p* < 0.001; **: *p* < 0.01; *: *p* < 0.05.

Except for LSI and SLA, all leaf traits of *C. spectabilis* in the forest understory exhibited highly significant positive correlations with one another. In contrast, in exposed land, both LSI and SLA showed significant negative correlations with all other traits, and LA was negatively correlated with LSI, while most of the remaining trait pairs displayed highly significant positive correlations (Figure 2).

**Figure 3:**
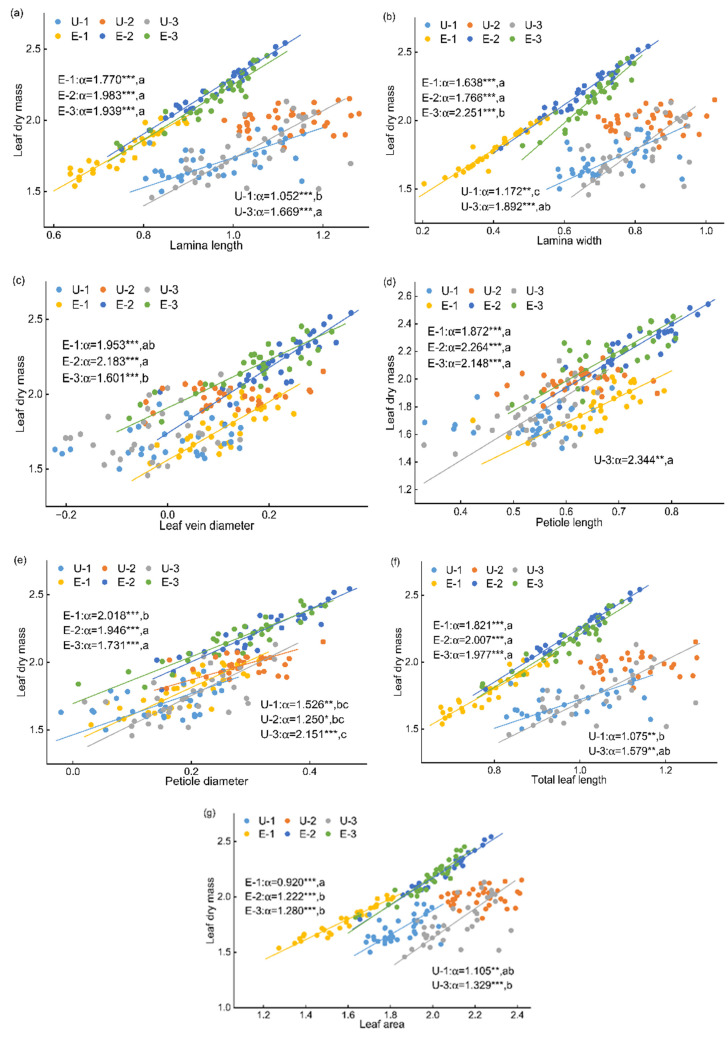
Allometric scaling exponents among leaf dry mass and main leaf traits (log_10_-transformed) of *Crotalaria spectabilis* in different habitats and canopy positions. U-1, -2, -3 indicate upper, middle, and lower canopy position in understory habitat; E-1, -2, -3 indicate upper, middle, and lower canopy position in the exposed land habitat. Regression lines are shown only for significant relationships (*p* < 0.05). (**a**) Leaf dry mass vs. Lamina length; (**b**) Leaf dry mass vs. Lamina width; (**c**) Leaf dry mass vs. Leaf vein diameter; (**d**) Leaf dry mass vs. Petiole length; (**e**) Leaf dry mass vs. Petiole diameter; (**f**) Leaf dry mass vs. Total leaf length; (**g**) Leaf dry mass vs. Leaf area. Different lowercase letters indicate significant differences among all groups (* *p* < 0.05, ** *p* < 0.01, *** *p* < 0.001).

**Figure 4:**
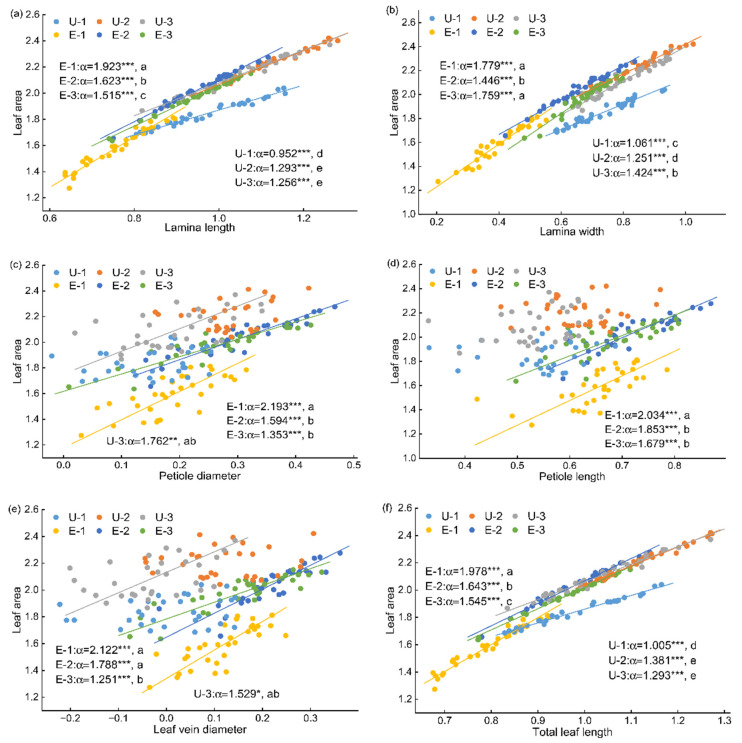
Allometric scaling exponents among leaf area and main leaf traits (log_10_-transformed) of *Crotalaria spectabilis* in different habitats and canopy positions. U-1, -2, -3 indicate upper, middle, and lower canopy position in understory habitat; E-1, -2, -3 indicate upper, middle, and lower canopy position in the exposed land habitat. Regression lines are shown only for significant relationships (*p* < 0.05). (**a**) Leaf area vs. Lamina length; (**b**) Leaf area vs. Lamina width; (**c**) Leaf area vs. Petiole diameter; (**d**) Leaf area vs. Petiole length; (**e**) Leaf area vs. Leaf vein diameter; (**f**) Leaf area vs. Total leaf length. Different lowercase letters indicate significant differences among all groups (* *p* < 0.05, ** *p* < 0.01, *** *p* < 0.001).

Allometric scaling analyses based on seven panel datasets showed that leaf allocation patterns differed markedly between the two habitats (Figure 3 and Figure 4). Overall, the goodness of fit among trait pairs was consistently higher in exposed land than in the understory, with *R*^2^ values ranging from 0.473 to 0.993 in exposed land, whereas those in the understory varied widely (0.154–0.982). For relationships between LM and the seven measured traits, significant allometric scaling was detected for most trait pairs, except for LM–LL, LM–LW, LM–TLL, and LM–LA in U2. Regression analyses between the seven measured traits and LA further indicated (Figure 4; Table S1) that, except for LA–PD and LA–LVD in U1 and U2, as well as LA–PL in both U1, U2 and U3, all other trait–LA relationships exhibited highly significant allometric scaling. Overall, allometric relationships among traits were more tightly fitted in exposed land than in the understory. Because leaves in the understory were generally smaller (i.e., lower LM), the scatterplots clearly separated the two habitats. Taking the LM–LL relationship as an example, slopes for the three canopy positions in exposed land were all significantly higher than those for U1 and U3, and except for U2, which showed isometric scaling, all other relationships exhibited hyperallometric scaling, indicating that the rate of increase in LM was significantly greater than that of LL. However, different allometric patterns were observed for LL–LW and LVD–PD relationships (Table S1). For instance, slopes of the LL–LW relationship across the three canopy positions in the understory were significantly higher than those in E1 and E2, indicating that LL increased more rapidly with LW in the understory than in exposed land, despite the significantly higher LSI observed in exposed land.

When data from the three canopy positions within each habitat were pooled for comparative analysis, allometric scaling relationships were detected between LM and each of the seven measured traits (Figures S1 and S2). Except for the LM–LW and LM–LA relationship, which showed no significant difference between habitats, the allometric scaling exponents for the remaining five trait pairs were significantly higher in exposed land than in the understory. This indicates that, with increasing morphological trait values, LM increased more rapidly in exposed land than in the understory (Figure S1). Except for the LA–PD and LA–LW relationship, which showed no significant difference between habitats, the allometric scaling exponents for the remaining four trait pairs were significantly higher in exposed land than in the understory (Figure S2).

### 2.3. Overall Differences in Leaf Traits Between Habitats

**Figure 5:**
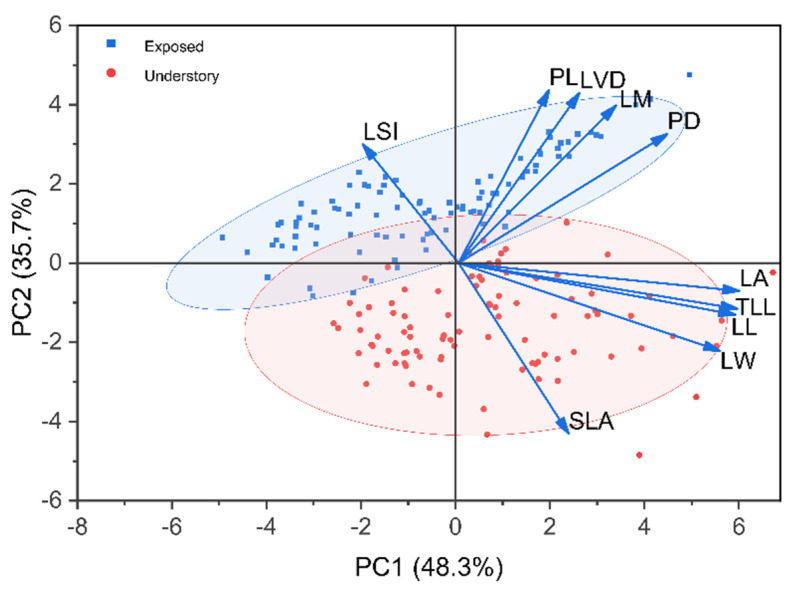
Principal Component Analysis indicating overall difference in leaf traits of *C. spectabilis* in different habitats based on trait matrix.

Principal component analysis (PCA) based on the matrix of ten leaf traits explained 84% of the total variance (Figure 5). Samples from the forest understory were mainly distributed in the lower part of the PCA ordination space, corresponding to broader leaves characterized by higher LL, LW, TLL, and LA, but lower LSI, PL, PD, LVD, and LM. In contrast, plants from exposed land were positioned in the upper part of the ordination space, corresponding to smaller leaves but higher values of LSI and LM. Within the same habitat, differences among the three canopy positions were most pronounced between E1 and E2/E3, with E1 exhibiting the highest LSI and the smallest leaf morphology (Figure S3).

**Figure 6:**
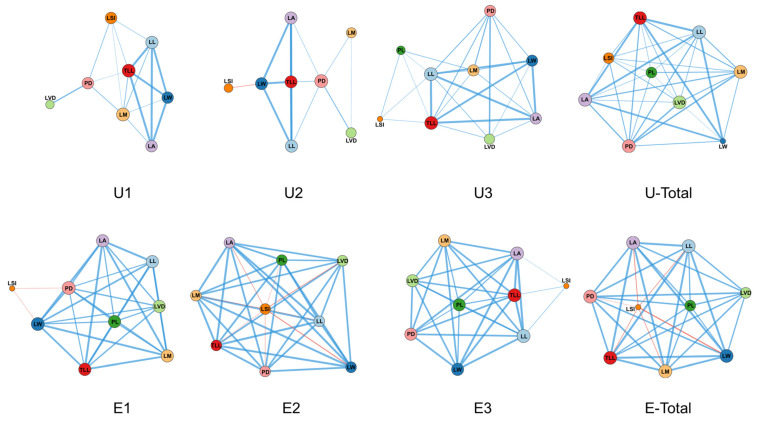
Plant trait networks (PTNs) of Crotalaria spectabilis across different habitats and canopy positions. Sky-blue lines indicate positive correlations, whereas red lines indicate negative correlations; line thickness represents the strength of the correlation coefficients. U1, U2, and U3 represent the upper, middle, and lower canopy positions in the forest understory, respectively, whereas E1, E2, and E3 represent the upper, middle, and lower canopy positions in exposed land. Total denotes pooled data across canopy positions within each habitat.

Plant trait network (PTN) analyses based on samples shown in the nine panels for the two habitats revealed pronounced differences in network structure (Figure 6). In exposed land, most leaf traits exhibited higher node degree values than those in the forest understory. At the whole-network level (Table S2), clustering coefficient (0.922–1), number of edges (30–36), and edge density (0.833–1) across canopy positions were consistently higher in exposed land than in the corresponding understory canopy positions (edges: 13–26; edge density: 0.361–0.722). In addition, network diameter and average path length were generally higher in exposed land. PTN analyses further indicated that correlations among leaf morphological traits in the understory were overall weaker and the network structure was relatively loose, whereas trait networks in exposed land were more tightly connected. Based on node centrality metrics, core traits differed markedly between habitats across canopy positions: in the understory, the core traits in the upper, middle, and lower canopy positions were LM, PD, and LL, respectively, whereas in exposed land the core traits were PD, LSI, and LA. When samples from the three canopy positions were pooled within each habitat, the core trait shifted to LVD in the understory and to PD in exposed land, indicating persistent differences between the two habitats.

## 3. Discussion

### 3.1. Habitat-Related Differences in Leaf Traits

Leaf functional traits are closely linked to plant biomass, growth strategies, and the acquisition and utilization of resources, and variation in functional traits reflects different survival strategies adopted by plants in response to environmental change [23]. In the present study, environmental differences between the forest understory and exposed land were mainly reflected in light intensity, temperature, and soil moisture, among which light availability is most likely the dominant factor. Light conditions strongly influence leaf functional traits [24], and as the primary photosynthetic organ, leaves are particularly sensitive to changes in irradiance [25]. Consistent with this finding, leaf morphology differed significantly between the two habitats in the present study: leaves in the understory had greater leaf length and width than those in exposed land, indicating that the higher irradiance in open habitats without shading constrains leaf area expansion but increases individual leaf mass. Leaf size is an important trait within the leaf economics spectrum [26], and when combined with statistical results for LA, it is evident that LA varied significantly among habitats and canopy positions. Two-way ANOVA showed that habitat, canopy position, and their interaction all had significant effects on LL (Table 2), suggesting that differences in light environment are the primary drivers of variation in lamina length. From the perspective of trait variability, the coefficient of variation (CV) of leaf area was generally higher in the understory than in exposed land, reaching its maximum in the lower canopy (Table 1). This pattern indicates that under low-light conditions and greater spatial heterogeneity in the understory, lamina length exhibits higher plasticity. Such elevated trait variability may allow individuals to flexibly adjust leaf size across microhabitats, thereby enhancing the capture of limited light resources. In contrast, the lower CV of leaf area observed in exposed land reflects stronger environmental filtering and functional constraints on leaf size under high irradiance and potential water stress. Shading is common among plants occupying overlapping niches, and the forest understory is characterized by a certain degree of canopy closure, resulting in photosynthetically active radiation levels that are lower than those in exposed land [27]. Plants often adapt to low-light environments by increasing light interception area to capture more light resources [28]. Studies examining leaf traits under different light and nutrient conditions have shown that shading significantly increases leaf area [29]. Under otherwise similar environmental conditions, prolonged shading has also been shown to reduce leaf area duration and net assimilation rate [30].

Lower LSI values in the forest understory indicate that leaves tend to be broader and more rounded, suggesting an adaptive strategy in which plants enhance light acquisition efficiency by adjusting leaf shape under low-light conditions. In contrast, the higher LSI observed in exposed land indicates that leaves are more elongated, a morphology that can help reduce transpirational water loss under high irradiance and potential water stress. These patterns are consistent with well-established ecological observations: plants inhabiting arid environments typically possess smaller, thicker, and more elongated leaves (sometimes even needle-like), whereas plants growing in humid environments generally develop larger, thinner, and broader leaves [31]. Therefore, although belonging to the same species, *C. spectabilis* exhibits pronounced plasticity in leaf traits when adapting to contrasting environments, highlighting its strong ecological resilience.

Leaf dry mass reflects a plant’s capacity for resource utilization and is positively associated with its ability to withstand external environmental stress [32]; it also represents the plant’s capacity for nutrient retention [15]. Leaf dry mass per unit area was lower in the understory than in exposed land, meaning that plants in the understory exhibited higher SLA, while those in exposed land showed lower SLA. Tree species in sandy habitats with higher light availability had higher SLA and lower leaf thickness than those in forest habitats [33]. Consistent patterns in SLA of woody plants across contrasting light environments have been documented [15]. These results suggest that while plants increase leaf area to adapt to low-light conditions, they also enhance light-use efficiency and reduce resource demand, resulting in lower dry matter accumulation [34].

Leaf veins provide biomechanical support for leaves and are responsible for the transport of water and nutrients during plant growth; thus, vein diameter can reflect differences in leaf hydraulic transport capacity [35,36]. The number and diameter of veins and xylem conduits are generally coordinated with leaf area, and leaf hydraulic capacity is positively correlated with leaf area [37,38]. Studies of leaf biomechanics demonstrate that leaves with larger areas tend to invest more in the midrib, resulting in larger vein diameters [39]; however, this pattern was not consistent with the results of the present study. Plants tend to adopt different vein growth strategies under contrasting water conditions, leading to variation in vein traits across habitats characterized by heterogeneous resource distribution [40]. *C. spectabilis* in the exposed land habitat with lower soil moisture in the present study may enhance the amount and rate of water transport by increasing vein diameter [41]. In addition, plants growing in exposed land exhibited longer and thicker petioles than those in the understory, which may be related to differences in individual leaf mass between the two habitats. Thicker petioles and veins confer greater mechanical support to leave [35].

The petiole serves as the transport pathway for water, nutrients, and assimilates between the stem and the leaf, while also providing mechanical support to the leaf; thus, petioles are closely linked to leaf biomechanics and growth and development [42]. Generally, larger leaves require longer petioles to extend the leaf blade and reduce mutual shading, and heavier leaves demand greater mechanical support from the petiole [43]. In the present study, *C. spectabilis* growing in the low-light understory produced large leaves with relatively short petioles, whereas plants in exposed land exhibited longer petioles. This pattern may help reduce mutual shading and is consistent with observations that fast-growing tropical plants with large leaves often have short petioles, reflecting trade-offs between leaf and petiole traits [44]. Studies of petiole–leaf trade-offs across different plant functional types indicate that the strategy of elongating petioles to adapt to low-light conditions may not be applicable to herbaceous species [45]. Instead, increasing chlorophyll content per unit leaf area [46] or enhancing photosynthetic activity [47] may represent the primary strategies by which herbaceous plants adapt to shaded understory environments. Plants often exhibit plasticity in leaf shape and structure in response to environmental conditions [48,49]. Taken together, variation in leaf morphological traits of *C. spectabilis* across habitats reflects a high degree of adaptive plasticity in response to environmental heterogeneity. From a practical production perspective, planting orientation is important when *C. spectabilis* is intercropped with taller crops. East–west row orientation may result in persistent shading, which is unfavorable for organic matter accumulation and efficient nitrogen fixation. Therefore, north–south row orientation is recommended to promote soil nutrient recovery.

### 3.2. Effects of Canopy Position on Leaf Traits

Leaves are the fundamental structural and functional units of plants, serving as the primary organs of photosynthesis and as key energy converters for primary producers in ecosystems. Leaf trait characteristics directly influence basic plant behaviors and functions and are closely associated with plant biomass as well as the efficiency of resource acquisition and utilization. These traits reflect the survival strategies that plants have evolved in response to environmental variation [50]. Previous studies have shown that most morphological traits of upper-canopy leaves in camphor trees (*Cinnamomum camphora*) are greater than those of lower-canopy leaves. The differentiation between upper- and lower-canopy leaves in trees results from the combined effects of genetic regulation (e.g., genes associated with heterophylly) and environmental pressures such as light availability, water conditions, and wind. In the present study, canopy position exerted highly significant effects on leaf trait variation under both forest understory and exposed land habitats (*p* < 0.01), and significant interactions between habitat and canopy position were also detected. These findings are consistent with those reported that most leaf functional traits of individual trees differed significantly across canopy heights [51].

In the forest understory, leaves from the middle canopy position exhibited significantly higher LL, PL, LVD, PD and LA than those from both the upper and lower canopy positions. Within the understory, LA of middle-canopy leaves was significantly greater than that of upper- and lower-canopy leaves, indicating that under low-light and spatially heterogeneous light conditions, middle-canopy leaves maximize interception of limited light resources by expanding leaf area. This finding is consistent with previous studies [52] and reflects the fine-scale regulation of light-use efficiency across canopy heights in understory plants. Leaves in the upper canopy were also relatively small, likely because light availability in the understory is generally limited and, for actively growing plants, upper-canopy leaves tend to be smaller and less effective in light capture. Consequently, leaf trait values in the middle canopy were higher than those in both the upper and lower canopy positions. SLA, an important indicator of a plant’s ability to deploy photosynthetic surface area per unit biomass investment, also differed significantly among habitats and canopy positions. SLA was significantly higher in the understory than in exposed land. This pattern suggests that understory plants tend to reduce structural investment per unit leaf area, constructing a larger photosynthetic surface at a lower dry-mass cost to enhance light-use efficiency, consistent with a more resource-conservative strategy. In contrast, lower SLA values in exposed land indicate higher dry matter content and greater structural strength of leaves, which may enhance tolerance to high irradiance, drought, and mechanical stress. Leaf vein diameter and petiole traits were generally more developed in exposed land than in the understory, further indicating that leaves in exposed land adopt a more conservative strategy by increasing structural investment to maintain hydraulic transport capacity and physiological stability, rather than relying solely on leaf area expansion. The vertical distribution patterns of leaf traits in exposed land differed markedly from those in the understory: LL, LW, and TLL were lowest in the upper canopy, with no significant differences between the middle and lower canopy positions, whereas LSI showed a decreasing trend from the upper to the lower canopy. However, a study of heteromorphic leaves in *Populus diversifolia* found that LSI decreased with increasing canopy height [52], which contrasts with our results. This discrepancy may be attributable to differences in life forms between woody and herbaceous plants. Herbaceous and woody species exhibit distinct leaf economic strategies [53], and leaf traits in herbaceous plants are generally more sensitive to phenological changes [54], potentially leading to contrasting patterns of leaf-shape variation compared with trees.

### 3.3. Effects of Habitat and Canopy Position on Covariation Patterns Among Leaf Traits

Correlation analyses showed that relationships among leaf traits differed markedly between habitats [36]. Vein traits exhibited pronounced differences in correlation structure between the two habitats. In exposed land, vein diameter was significantly positively correlated with leaf length, leaf width, and LA, indicating that under high irradiance and potential water stress, the venation system must scale synchronously with leaf size to maintain efficient transport of water and nutrients [55]. Studies on leaf venation traits have similarly shown significant positive relationships between vein diameter and leaf length and width [56], which is consistent with the venation patterns observed in exposed land in the present study. Together, these differences in trait–trait correlations between habitats demonstrate that contrasting light environments exert significant influences on the relationships among leaf traits [57]. LSI showed weak correlations with other traits in the understory, whereas in exposed land it was strongly and negatively correlated with most other traits, reflecting pronounced trade-off patterns. Under low-light conditions, leaf area expansion relies more on reducing structural investment per unit area to enhance light-use efficiency [58]. In exposed land, LA was significantly positively correlated with leaf length, leaf width, and total leaf length, and the negative correlations between SLA and traits such as leaf width and vein diameter were more pronounced. These results reflect a clearer trade-off between leaf area expansion and structural investment under high-light conditions [47].

Among the significant allometric relationships observed in both habitats, the allometric exponents differed significantly between habitats. The differences in allometric relationships among traits followed a consistent pattern across habitats, with all allometric exponents being higher in exposed land than in the understory. This pattern suggests that under higher light availability, leaf traits respond more rapidly to changes in scale, whereas low-light conditions tend to constrain coordinated growth among traits [59]. As leaf length increased, the rate of increase in total leaf length was generally slower, and this trend was more pronounced in the understory, indicating that low-light environments may suppress the expansion capacity of leaf structural components. Leaf length and leaf width jointly determine leaf shape [5]. In exposed land, leaf width increased significantly faster than leaf length, resulting in more pronounced lateral expansion as leaf size increased, thereby facilitating a rapid increase in LA. The rate at which LM increased relative to other leaf traits was significantly higher in exposed land than in the understory, indicating that high-light conditions strongly promoted rapid dry matter accumulation in leaves. A higher rate of dry matter accumulation enhances leaf structural stability and stress resistance, allowing leaves to maintain high functional safety while expanding leaf area, thereby improving adaptation to high irradiance and potential water stress [60]. Under the lower light conditions of the understory, growth relationships among leaf traits tended to become decoupled, and leaf area expansion was achieved mainly through adjustments in morphological proportions and size-related traits. By contrast, higher light availability in exposed land promoted coordinated growth among leaf traits, enabling leaf length, leaf width, leaf area, and structural traits to remain more tightly coupled during expansion. This resulted in a more integrated pattern of leaf morphological construction. Overall, contrasting light environments profoundly influence the coordination among leaf traits and their ecological adaptive strategies by regulating allometric growth relationships [6,7]. Plant leaf traits are not only influenced by habitat and canopy position, but also by leaf age—a critical intrinsic factor [61]. Leaf age undergoes dynamic changes throughout the entire lifespan of a leaf, from emergence to maturity and senescence [62]. Additionally, it interacts complexly with environmental factors such as light, potentially confounding the independent attribution of canopy position effects in this study.

In the two-dimensional PCA ordination based on the leaf-trait matrix, plants from the two habitats exhibited a separation trend in their distribution, despite some overlap. Individuals from the exposed land were mainly clustered in the upper part of the ordination space, whereas *Crotalaria spectabilis* individuals from the understory were predominantly distributed in the lower part and occupied a broader range. This differential clustering is consistent with the influence of strong habitat heterogeneity on trait expression, promoting divergence along the primary PCA axes [63]. Examination of trait loadings on the principal component axes revealed that LA plays a key role in distinguishing leaf trait syndromes between the two habitats and reflects substantial differences in the scale of light capture under contrasting light environments. PTN analysis further showed that the exposed-land habitat exhibited higher clustering coefficients and edge density, indicating stronger coordination among leaf traits [64]. In contrast, the understory leaf trait network had fewer edges and lower edge density, suggesting greater independence among traits [12,65]. In exposed land, all nine leaf traits were closely interconnected, with multiple linkages, trade-offs, and mutual constraints among traits, which is consistent with the relatively narrow distribution of exposed-land samples in the PCA ordination. The traits governing phenotypic expression and functional regulation of *C. spectabilis* leaves differ across light environments. Previous studies have similarly shown that PTN structure within a species can vary under different environmental conditions. For example, in *Alyssum linifolium*, PTN relationships differed among nutrient addition treatments, with phosphorus addition significantly reducing network complexity and trait connectivity [12]. In woody species such as juvenile *Malus sieversii*, nutrient addition increased PTN tightness among traits including basal twig diameter, stem biomass, and whole-branch biomass [66]. Collectively, these studies demonstrate that functional trait relationships within a species exhibit varying degrees of plasticity in response to environmental variation. From a functional perspective, leaf length, leaf width, leaf area, total leaf length, and leaf shape index primarily contribute to the construction of external leaf morphology and the spatial configuration for light capture, whereas leaf dry mass, petiole diameter, and vein diameter are more closely associated with resource transport and structural maintenance [9,63]. In the exposed-land habitat, tighter PTN connectivity among traits enables coordinated variation, which helps enhance structural stability and functional safety while expanding leaf area, thereby improving plant adaptation to high irradiance and potential water stress. Habitat shifts thus not only strongly influence the expression of individual functional traits but also reshape the positional roles of key traits such as leaf area within PTNs, ultimately altering overall patterns of trait coordination. Plants with stronger trait associations and higher degrees of functional integration tend to exhibit greater adaptive capacity to environmental change. However, although key microclimatic variables (PPFD, air temperature, relative humidity, and soil moisture) were measured simultaneously in both habitats, other potentially important environmental factors (e.g., soil nutrient availability and detailed canopy structure/openness) were not quantified. Future studies incorporating these variables and longer-term monitoring would further strengthen habitat characterization and improve mechanistic interpretation.

## 4. Materials and Methods

### 4.1. Study Area

Sampling was conducted in two habitat types within a *Lagerstroemia indica* plantation in Anqing, Anhui Province, China—an understory and an adjacent exposed land—located ~40 m apart (30.62° N, 117.00° E). The mean height of the *L. indica* trees in the garden was 3.26 m. Beyond the planted *L. indica*, the ground layer supported diverse herbs, including *Daucus carota*, *Setaria viridis*, *Vicia sepium*, and various bryophytes. Soils were classified as yellow-brown and yellow-cinnamon types. The soil organic matter, total nitrogen, and total phosphorus were 25.43, 1.56, and 0.82 g kg^−1^, respectively; available nitrogen and available phosphorus were 76.49 and 23.37 mg kg^−1^ [7]. Overall soil fertility was moderate, and soil pH was 7.8 (slightly alkaline). Microclimatic variables were measured simultaneously in the two habitats (understory and exposed land) around midday on the same day. Measurements were conducted at the herbaceous canopy height using a five-point sampling scheme in each habitat (*n* = 5 sampling points per habitat). At each sampling point, measurements were repeated three times at 10 min intervals, and the three readings were averaged to obtain a point-level value. Light intensity was recorded as illuminance (lux) using a handheld lux meter (Testo 540, Testo SE & Co. KGaA, Titisee-Neustadt, Germany) at the herbaceous canopy height. Illuminance values were converted to photosynthetic photon flux density (PPFD, μmol m^−2^ s^−1^) using the sunlight conversion factor: PPFD = lux × 0.0185. The lux-to-PPFD conversion depends on the spectral distribution of light; thus, converted PPFD values represent an approximation under the assumed daylight conditions. Air temperature and relative humidity were measured using a portable thermo-hygrometer (Testo 605i, Testo SE & Co. KGaA, Titisee-Neustadt, Germany) at the herbaceous canopy height following the same replication schedule. Soil moisture was measured using a portable soil moisture meter (TZS-2X-G, Top Cloud-Agri, Hangzhou, China) in the 0–10 cm soil layer at the same sampling points; three repeated readings were taken at 10 min intervals and averaged for each point. Values presented in Table 3 represent the mean ± SD calculated across the five point-level averages (*n* = 5) for each habitat. At midday, key microclimatic variables differed markedly between habitats: air temperature and irradiance were higher in the exposed land, whereas relative humidity and soil water content were higher in the understory (Table 3). Here, the “Exposed land” denotes a leveled area without planted woody vegetation where herbaceous species occur naturally. Apart from these contrasts, herbaceous plant density was higher in the understory, while the two habitats shared the same soil types.

### 4.2. Methods

#### 4.2.1. Plant Sampling and Leaf-Trait Measurements

We conducted the experiment during peak flowering in mid-November. In each habitat—the crape myrtle understory and the adjacent exposed land—we established six 5 m × 5 m plots. Within each plot, we randomly selected 5–6 reproductive individuals (bearing both flowers and fruits). In total, 36 individuals were sampled in the understory and 32 in the exposed land. Mean plant height was 68 cm in the understory and 85 cm in the exposed land [7].

For each individual plant, branches and leaves were divided into three vertical layers—upper, middle, and lower—according to canopy height [67]. At each canopy position, three healthy and intact leaves were selected from the south-facing side of each plant and placed in an insulated container with ice packs for rapid transport to the laboratory. Leaf lamina length (LL, cm) and lamina width (LW, cm) were measured using a ruler. The diameter of the midrib at the central portion of the leaf (leaf vein diameter, LVD, mm), the diameter at the midpoint of the petiole (petiole diameter, PD, mm), and petiole length (PL, mm) were measured using a digital vernier caliper. For each leaf, measurements of LVD and PD were taken twice and averaged for subsequent analyses. All measurements were conducted at standardized positions on each leaf. Leaves were then scanned using a flatbed scanner, and leaf area (LA, cm^2^) was calculated using ImageJ software (v. 1.54g; National Institutes of Health, Bethesda, MD, USA) Finally, leaf samples were oven-dried at 70 °C to constant mass and weighed with an analytical balance (precision 0.1 mg) to obtain individual leaf dry mass (LM, mg). The leaf-shape index (LSI) was calculated as the ratio of LL to LW [68] and total leaf length (TLL, cm) was calculated as the sum of LL and PL. In total, 108 and 96 valid leaf samples were obtained from the forest understory and exposed land habitats, respectively.

#### 4.2.2. Statistical Analyses

For each plant × canopy position, trait values were averaged across the three leaves; these plant-level means were used for all subsequent analyses (replication unit: individual plant). We summarized nine leaf traits—LL, LW, LVD, PL, PD, LSI, LM, TLL and LA—using descriptive statistics. We then applied two-way ANOVA to test the main effects of habitat (understory vs. exposed land) and canopy position (upper, middle, lower) and their interaction. To compare trait means across the six habitat × canopy groups, we additionally conducted one-way ANOVAs followed by post hoc multiple comparisons. Pairwise associations among traits were evaluated using Pearson correlation coefficients. Routine data handling was carried out in Excel 2019, and descriptive statistics, correlation analyses, and ANOVAs were performed in SPSS 20.0. For clarity, canopy positions within each habitat were coded as U1 (upper canopy), U2 (middle canopy), and U3 (lower canopy) for the understory, and E1 (upper canopy), E2 (middle canopy), and E3 (lower canopy) for exposed land.

We characterized pairwise allometric scaling relationships among leaf traits using the power function *Y* = *b*·*X^α^* [28], where *X* and *Y* are leaf traits of *C. spectabilis*, *α* is the allometric scaling exponent, and *b* is a constant. When *α* = 1, the relationship is isometric, indicating proportional change in the dependent variable with the independent variable; when *α* ≠ 1, the relationship is allometric, with *α* > 1 denoting hyperallometry and *α* < 1 denoting hypoallometry. After log-transforming both variables, we estimated *α* (the log–log slope), its 95% confidence interval, and the coefficient of determination (*R*^2^) using type-II reduced major axis (RMA) regression [28]. Models were fitted separately for the two habitats and for each of the six habitat × canopy groups, followed by multiple comparisons of exponents among groups. Allometric scaling analyses and tests of isometry (H_0_: *α* = 1) were performed using the “smatr” package in R 4.5.1.

The principal component analysis (PCA) was employed to analyze the leaf-trait matrix comprising all samples from both habitats to assess overall, trait-based differentiation between habitats [69]. To characterize multivariate trait interrelationships, we constructed plant trait networks (PTNs) separately for each habitat and canopy position and quantified their network topology [63]. Traits were treated as nodes, and edges were retained only for trait pairs showing significant Pearson correlations (*p* < 0.05) and exceeding an effect-size threshold (|r| > 0.3) to reduce spurious associations (replication unit: individual plant; *n* = 36 (understory) and *n* = 32 (exposed land)) [12]. Core traits were identified using node-level metrics (degree, betweenness centrality, and clustering coefficient), and traits consistently ranking highest across these indices were considered core traits. PTN edges represent statistical associations among traits rather than direct causal relationships.

## 5. Conclusions

This study systematically examined the effects of habitat conditions and canopy position on the environmental plasticity of leaf functional traits in the large dicotyledonous leguminous herb *C. spectabilis*. The results showed that, compared with canopy position, habitat conditions exerted a stronger influence on leaf traits, and significant interactions between habitat and canopy position were detected. Leaves in the exposed-land habitat exhibited higher individual leaf dry mass, larger vein diameter, and longer petioles, with more elongated leaf shapes and higher coordination and stability among leaf traits, reflecting an adaptive strategy characterized by greater structural investment. In contrast, leaves in the understory were generally larger but lighter, with higher specific leaf area, and showed relatively decoupled trait relationships, indicating a strategy primarily aimed at enhancing light resource acquisition and use efficiency. In addition, leaves at the middle canopy position exhibited the highest values for most traits in both habitats, indicating that pronounced canopy effects also occur in large herbaceous plants. Overall, leaf traits of *C. spectabilis* and their interrelationships exhibit high plasticity in response to habitat heterogeneity.

From an applied perspective, these findings provide a scientific reference for the management and practical utilization of *C. spectabilis* under contrasting habitat conditions.

## Figures and Tables

**Table 1:** Coefficients of variation in leaf traits of *Crotalaria spectabilis* in different habitats and canopy positions.

Habitat	Canopy Position	LL	LW	LVD	PL	PD	TLL	LM	LSI	LA	SLA
Understory	Upper	0.252	0.229	0.218	0.167	0.161	0.245	0.276	0.096	0.241	0.272
	Middle	0.203	0.209	0.213	0.163	0.130	0.193	0.180	0.073	0.260	0.299
	Lower	0.261	0.229	0.208	0.156	0.182	0.259	0.448	0.097	0.323	0.418
Exposed land	Upper	0.179	0.192	0.160	0.156	0.155	0.174	0.316	0.093	0.328	0.098
	Middle	0.192	0.214	0.170	0.174	0.197	0.190	0.380	0.053	0.295	0.098
	Lower	0.176	0.157	0.216	0.170	0.201	0.173	0.357	0.056	0.249	0.145

LL: Lamina length; LW: Lamina width; LVD: Leaf vein diameter; PL: Petiole length; PD: Petiole diameter; TLL: Total leaf length; LM: Leaf dry mass; LSI: Leaf-shape index; LA: Leaf area; SLA: Specific leaf area.

**Table 2:** Effects of different habitats, canopy positions, and their interactions on leaf traits of *Crotalaria spectabilis* (*F*-value).

Source of Variance	LL	LW	LVD	PL	PD	TLL	LM	LSI	LA	SLA
Habitat	106.19 ***	195.45 ***	100.67 ***	126.21 ***	18.46 ***	77.22 ***	121.28 ***	160.27 ***	78.74 ***	290.33 ***
Position	47.41 ***	50.76 ***	31.87 ***	19.15 ***	45.66 ***	44.68 ***	72.67 ***	40.94 ***	132.27 ***	17.11 ***
Habitat * Position	5.14 **	5.41 **	3.53 *	0.82	2.07	5.70**	19.62 ***	43.64 ***	2.63	13.22 ***

Asterisks indicate the significance of the F-tests from the two-way ANOVA (Habitat, Position, and Habitat × Position): * *p* < 0.05, ** *p* < 0.01, *** *p* < 0.001.

**Table 3:** Individual density and ecological factors in the two habitats.

Habitat	Density (Ind m^−2^)	Air Temperature (°C)	Relative Humidity (%)	Photon Flux Density (μmol m^−2^ s^−1^)	Soil Moisture (%)
Understory	4.12 ± 0.250 a	27.29 ± 0.659 b	48.88 ± 1.025 a	784.68 ± 48.96 b	6.29 ± 0.21 a
Exposed land	4.75 ± 0.227 a	34.88 ± 0.745 a	38.25 ± 0.818 b	3222.22 ± 88.13 a	5.56 ± 0.36 b

Letters denote significant differences (*p* < 0.05).

## Data Availability

The datasets generated and/or analyzed during the current study are available from the corresponding authors on reasonable request due to privacy restrictions.

## Supplementary Materials

The following are available online at https://www.mdpi.com/article/10.3390/plants15030492/s1, Table S1: Allometric scaling exponents and isometric test among leaf traits (log10-transformed) of Crotalaria spectabilis in different habitats and canopy positions; Table S2: Parameters of leaf trait networks of Crotalaria spectabilis in different habitats and canopy positions; Figure S1. Allometric scaling exponents among leaf mass and main leaf traits (log10-transformed) of Crotalaria spectabilis in different habitats. U: understory habitat, E: exposed land habitat. Letters denote significant differences (* *p* < 0.05, ** *p* < 0.01, *** *p* < 0.001); Figure S2. Allometric scaling exponents among leaf area and main leaf traits (log10-transformed) of Crotalaria spectabilis in different habitats. U: understory habitat, E: exposed land habitat. Letters denote significant differences (* *p* < 0.05, ** *p* < 0.01, *** *p* < 0.001); Figure S3. Principal Component Analysis indicating overall difference in leaf traits of Crotalaria spectabilis in two habitats and three canopy positions based on trait matrix. U1 (upper canopy), U2 (middle canopy), and U3 (lower canopy) for the understory, and E1 (upper canopy), E2 (middle canopy), and E3 (lower canopy) for exposed land.
