# Supplementary material for: Habitat and Canopy Position Influence Leaf Traits and Trait-Associations of a Large-Sized Leguminous Herb (Crotalaria spectabilis)
Source: Plants (Basel). 2026 Feb 5;15(3):492. doi: 10.3390/plants15030492 (PMC12899842; doi:10.3390/plants15030492)
Supplement: Supplementary file 1 [file plants-15-00492-s001.zip › plants-4090959-supplementary.pdf]

Supplementary material

Table S1 Allometric scaling exponents and isometric test among leaf traits (log_{10}-transformed) of *Crotalaria spectabilis* in different habitats and canopy positions.

Y	*X*	Habitat	Canopy position	Allometric scaling exponent					Isometric test	
				R^2	*P*	Slope	LowCI	UppCI	*F*	*P*
LM	LL	Understory	Up	0.315	0.000	1.052	0.791	1.398	0.127	0.723
			Md	0.064	0.136	0.915	0.657	1.274	0.288	0.595
			Lo	0.385	0.000	1.669	1.274	2.186	15.820	0.000
		Exposed land	Up	0.872	0.000	1.770	1.549	2.022	84.670	0.000
			Md	0.969	0.000	1.983	1.857	2.118	526.587	0.000
			Lo	0.875	0.000	1.939	1.699	2.211	121.084	0.000
	LW	Understory	Up	0.215	0.004	1.172	0.865	1.589	1.100	0.302
			Md	0.037	0.261	0.885	0.633	1.238	0.530	0.472
			Lo	0.355	0.000	1.892	1.435	2.494	24.470	0.000
		Exposed land	Up	0.926	0.000	1.638	1.480	1.812	107.172	0.000
			Md	0.950	0.000	1.766	1.625	1.920	215.814	0.000
			Lo	0.875	0.000	2.251	1.974	2.567	196.123	0.000
	LVD	Understory	Up	0.114	0.063	1.101	0.775	1.562	0.302	0.587
			Md	0.126	0.050	0.774	0.547	1.096	2.229	0.146
			Lo	0.096	0.089	1.866	1.311	2.658	14.206	0.001
		Exposed land	Up	0.593	0.000	1.953	1.543	2.472	38.283	0.000
			Md	0.713	0.000	2.183	1.790	2.663	77.677	0.000
			Lo	0.752	0.000	1.601	1.331	1.925	28.828	0.000
	PL	Understory	Up	0.053	0.211	1.426	0.993	2.047	4.020	0.054
			Md	0.061	0.180	1.013	0.707	1.452	0.005	0.942
			Lo	0.282	0.002	2.344	1.708	3.217	37.142	0.000
		Exposed land	Up	0.473	0.000	1.872	1.432	2.445	25.467	0.000
			Md	0.818	0.000	2.264	1.932	2.653	136.773	0.000
			Lo	0.552	0.000	2.148	1.678	2.750	47.386	0.000
	PD	Understory	Up	0.216	0.008	1.526	1.097	2.124	7.025	0.013
			Md	0.154	0.029	1.250	0.887	1.760	1.732	0.198
			Lo	0.400	0.000	2.151	1.609	2.874	34.342	0.000
		Exposed land	Up	0.608	0.000	2.018	1.601	2.544	44.338	0.000
			Md	0.851	0.000	1.946	1.687	2.246	103.577	0.000
			Lo	0.843	0.000	1.731	1.494	2.006	63.560	0.000
	TLL	Understory	Up	0.258	0.004	1.075	0.779	1.483	0.203	0.656
			Md	0.013	0.545	0.887	0.613	1.283	0.426	0.519
			Lo	0.288	0.002	1.579	1.152	2.164	9.096	0.005
		Exposed land	Up	0.880	0.000	1.821	1.600	2.071	100.588	0.000
			Md	0.970	0.000	2.007	1.883	2.140	576.766	0.000
			Lo	0.882	0.000	1.977	1.740	2.247	137.130	0.000
	LA	Understory	Up	0.279	0.001	1.105	0.825	1.479	0.471	0.497
			Md	0.054	0.173	0.708	0.507	0.987	4.475	0.042

			Lo	0.394	0.000	1.329	1.016	1.737	4.650	0.038
		Exposed land	Up	0.935	0.000	0.920	0.837	1.012	3.189	0.084
			Md	0.965	0.000	1.222	1.139	1.310	34.771	0.000
			Lo	0.867	0.000	1.280	1.117	1.466	13.972	0.001
LA	LL	Understory	Up	0.970	0.000	0.952	0.897	1.011	2.782	0.104
			Md	0.977	0.000	1.293	1.226	1.364	99.025	0.000
			Lo	0.977	0.000	1.256	1.192	1.324	79.224	0.000
		Exposed land	Up	0.951	0.000	1.923	1.771	2.088	301.416	0.000
			Md	0.987	0.000	1.623	1.557	1.693	605.071	0.000
			Lo	0.993	0.000	1.515	1.468	1.563	758.437	0.000
	LW	Understory	Up	0.954	0.000	1.061	0.984	1.143	2.577	0.118
			Md	0.958	0.000	1.251	1.164	1.344	40.830	0.000
			Lo	0.950	0.000	1.424	1.317	1.539	88.462	0.000
		Exposed land	Up	0.926	0.000	1.779	1.608	1.969	150.035	0.000
			Md	0.976	0.000	1.446	1.364	1.533	174.334	0.000
			Lo	0.942	0.000	1.759	1.608	1.924	183.373	0.000
	LVD	Understory	Up	0.038	0.293	1.029	0.715	1.482	0.025	0.874
			Md	0.003	0.754	1.205	0.832	1.746	1.025	0.320
			Lo	0.184	0.016	1.529	1.092	2.141	6.799	0.014
		Exposed land	Up	0.600	0.000	2.122	1.680	2.681	51.070	0.000
			Md	0.731	0.000	1.788	1.475	2.166	42.044	0.000
			Lo	0.752	0.000	1.251	1.040	1.505	6.153	0.019
	PL	Understory	Up	0.000	0.958	1.334	0.920	1.933	2.472	0.127
			Md	0.007	0.658	1.578	1.090	2.284	6.507	0.016
			Lo	0.119	0.057	1.920	1.354	2.723	16.120	0.000
		Exposed land	Up	0.489	0.000	2.034	1.563	2.647	34.889	0.000
			Md	0.779	0.000	1.853	1.557	2.207	58.575	0.000
			Lo	0.501	0.000	1.679	1.294	2.178	17.626	0.000
	PD	Understory	Up	0.071	0.146	1.428	0.998	2.043	4.131	0.051
			Md	0.096	0.089	1.946	1.366	2.772	16.457	0.000
			Lo	0.293	0.002	1.762	1.287	2.412	14.619	0.001
		Exposed land	Up	0.543	0.000	2.193	1.709	2.814	49.514	0.000
			Md	0.837	0.000	1.594	1.372	1.851	42.931	0.000
			Lo	0.818	0.000	1.353	1.155	1.585	15.494	0.000
	TLL	Understory	Up	0.980	0.000	1.005	0.953	1.061	0.040	0.843
			Md	0.982	0.000	1.381	1.313	1.453	174.365	0.000
			Lo	0.972	0.000	1.293	1.214	1.377	70.875	0.000
		Exposed land	Up	0.957	0.000	1.978	1.831	2.137	378.361	0.000
			Md	0.986	0.000	1.643	1.573	1.717	575.968	0.000
			Lo	0.993	0.000	1.545	1.497	1.594	842.010	0.000
LL	LW	Understory	Up	0.856	0.000	1.114	0.977	1.271	2.780	0.105
			Md	0.878	0.000	0.967	0.857	1.092	0.310	0.581
			Lo	0.869	0.000	1.133	0.999	1.286	4.081	0.051
		Exposed land	Up	0.791	0.000	0.926	0.781	1.097	0.859	0.362

			Md	0.953	0.000	0.891	0.822	0.966	8.560	0.006
			Lo	0.919	0.000	1.161	1.045	1.291	8.389	0.007
	LVD	Understory	Up	0.057	0.197	1.063	0.741	1.525	0.115	0.737
			Md	0.010	0.586	0.898	0.621	1.300	0.339	0.565
			Lo	0.204	0.011	1.210	0.868	1.688	1.341	0.256
		Exposed land	Up	0.569	0.000	1.104	0.866	1.406	0.680	0.416
			Md	0.726	0.000	1.101	0.907	1.337	1.021	0.320
			Lo	0.752	0.000	0.826	0.687	0.993	4.489	0.043
	PL	Understory	Up	0.001	0.865	-1.377	-1.996	-0.950	3.075	0.090
			Md	0.009	0.604	1.176	0.813	1.702	0.776	0.386
			Lo	0.161	0.025	1.520	1.081	2.138	6.426	0.017
		Exposed land	Up	0.432	0.000	1.058	0.802	1.396	0.167	0.686
			Md	0.805	0.000	1.142	0.969	1.345	2.715	0.110
			Lo	0.487	0.000	1.108	0.851	1.443	0.620	0.437
	PD	Understory	Up	0.104	0.076	1.474	1.037	2.097	5.129	0.031
			Md	0.136	0.041	1.450	1.026	2.050	4.857	0.036
			Lo	0.323	0.001	1.394	1.025	1.897	4.909	0.035
		Exposed land	Up	0.441	0.000	1.141	0.866	1.502	0.935	0.341
			Md	0.850	0.000	0.982	0.850	1.134	0.069	0.795
			Lo	0.820	0.000	0.893	0.763	1.046	2.139	0.154
	TLL	Understory	Up	0.999	0.000	1.038	1.028	1.048	64.827	0.000
			Md	0.999	0.000	1.029	1.019	1.040	31.936	0.000
			Lo	1.000	0.000	1.024	1.016	1.031	42.247	0.000
		Exposed land	Up	0.997	0.000	1.029	1.009	1.049	8.932	0.006
			Md	1.000	0.000	1.012	1.004	1.020	9.787	0.004
			Lo	0.999	0.000	1.020	1.007	1.033	10.599	0.003
LW	LVD	Understory	Up	0.020	0.452	0.979	0.678	1.414	0.014	0.908
			Md	0.000	0.916	0.998	0.688	1.446	0.000	0.989
			Lo	0.156	0.028	1.081	0.768	1.522	0.209	0.651
		Exposed land	Up	0.566	0.000	1.192	0.935	1.521	2.161	0.152
			Md	0.763	0.000	1.236	1.032	1.481	5.773	0.023
			Lo	0.745	0.000	0.711	0.590	0.858	14.190	0.001
	PL	Understory	Up	0.004	0.750	1.268	0.875	1.837	1.672	0.206
			Md	0.005	0.706	1.306	0.902	1.891	2.126	0.156
			Lo	0.059	0.187	1.358	0.947	1.947	2.976	0.095
		Exposed land	Up	0.490	0.000	1.143	0.878	1.487	1.053	0.313
			Md	0.778	0.000	1.282	1.076	1.527	8.484	0.007
			Lo	0.565	0.000	0.954	0.748	1.218	0.150	0.701
	PD	Understory	Up	0.038	0.295	1.358	0.943	1.954	2.904	0.099
			Md	0.066	0.163	1.611	1.125	2.307	7.607	0.010
			Lo	0.236	0.006	1.246	0.899	1.726	1.861	0.183
		Exposed land	Up	0.620	0.000	1.232	0.981	1.548	3.497	0.071
			Md	0.832	0.000	1.102	0.946	1.283	1.689	0.204
			Lo	0.815	0.000	0.769	0.656	0.902	11.454	0.002

	TLL	Understory	Up	0.906	0.000	0.956	0.851	1.073	0.634	0.432
			Md	0.919	0.000	1.143	1.026	1.273	6.416	0.017
			Lo	0.854	0.000	0.914	0.791	1.057	1.593	0.217
		Exposed land	Up	0.804	0.000	1.112	0.943	1.310	1.719	0.200
			Md	0.953	0.000	1.136	1.048	1.232	10.490	0.003
			Lo	0.926	0.000	0.878	0.794	0.972	6.911	0.013
LVD	PL	Understory	Up	0.022	0.427	-1.296	-1.871	-0.897	2.032	0.165
			Md	0.014	0.529	1.309	0.906	1.893	2.187	0.150
			Lo	0.001	0.873	1.256	0.867	1.820	1.536	0.225
		Exposed land	Up	0.469	0.000	0.958	0.733	1.254	0.102	0.751
			Md	0.564	0.000	1.037	0.812	1.323	0.090	0.766
			Lo	0.400	0.000	1.342	1.009	1.785	4.451	0.043
	PD	Understory	Up	0.478	0.000	1.387	1.058	1.819	6.153	0.019
			Md	0.315	0.001	1.615	1.185	2.200	10.497	0.003
			Lo	0.653	0.000	1.152	0.923	1.439	1.690	0.204
		Exposed land	Up	0.648	0.000	1.034	0.830	1.287	0.092	0.763
			Md	0.770	0.000	0.891	0.746	1.065	1.729	0.198
			Lo	0.817	0.000	1.082	0.923	1.268	1.008	0.323
	TLL	Understory	Up	0.055	0.203	0.977	0.680	1.401	0.017	0.896
			Md	0.011	0.575	1.146	0.792	1.658	0.547	0.465
			Lo	0.201	0.011	0.846	0.606	1.180	1.027	0.319
		Exposed land	Up	0.588	0.000	0.932	0.735	1.182	0.360	0.553
			Md	0.725	0.000	0.919	0.757	1.116	0.775	0.386
			Lo	0.753	0.000	1.235	1.027	1.485	5.497	0.026
PL	PD	Understory	Up	0.007	0.651	1.071	0.740	1.550	0.136	0.715
			Md	0.058	0.190	1.233	0.860	1.769	1.375	0.250
			Lo	0.110	0.068	0.917	0.646	1.303	0.243	0.625
		Exposed land	Up	0.592	0.000	1.078	0.852	1.365	0.419	0.522
			Md	0.772	0.000	0.860	0.720	1.027	3.022	0.092
			Lo	0.390	0.000	0.806	0.605	1.074	2.324	0.138
	TLL	Understory	Up	0.000	0.967	-0.754	-1.093	-0.520	2.380	0.134
			Md	0.015	0.507	0.875	0.606	1.265	0.526	0.474
			Lo	0.176	0.019	0.673	0.480	0.945	5.793	0.023
		Exposed land	Up	0.484	0.000	0.973	0.746	1.268	0.045	0.834
			Md	0.821	0.000	0.887	0.758	1.037	2.445	0.128
			Lo	0.520	0.000	0.920	0.713	1.188	0.432	0.516
PD	TLL	Understory	Up	0.106	0.073	0.704	0.495	1.001	4.163	0.051
			Md	0.140	0.038	0.710	0.502	1.002	4.128	0.051
			Lo	0.325	0.001	0.734	0.540	0.998	4.239	0.049
		Exposed land	Up	0.471	0.000	0.902	0.690	1.179	0.606	0.442
			Md	0.854	0.000	1.031	0.895	1.189	0.194	0.663
			Lo	0.818	0.000	1.142	0.975	1.338	2.920	0.098

Up: upper position, Md: middle position; Lo: lower position.

Table S2 Parameters of leaf trait networks of *Crotalaria spectabilis* in different habitats and canopy positions

Treatment	Trait	Node parameter			Network parameter				
		Degree	Betweenness centrality	Clustering coefficient	Clustering coefficient	Edges	Edge density	Average path length	Diameter
U-1	LL	5	0	0.800	0.750	17	0.472	0.723	1.625
	LW	4	0	1					
	LVD	1	0	0					
	PD	4	6	0.500					
	TLL	6	0	0.667					
	LM	6	11	0.667					
	LSI	4	3	0.833					
	LA	4	0	1					
U-2	LL	4	0	0.833	0.667	13	0.361	0.953	2.405
	LW	4	6	0.500					
	LVD	2	0	1					
	PD	5	13	0.400					
	TLL	4	0	0.833					
	LM	2	0	1					
	LSI	1	0	0					
	LA	4	6	0.833					
U-3	LL	8	7	0.643	0.8	26	0.722	0.737	1.413
	LW	6	1	0.933					
	LVD	5	1	1					
	PL	4	1	0.833					
	PD	6	0	0.933					
	TLL	8	0	0.643					
	LM	6	4	0.800					
	LSI	3	0	1					
	LA	6	0	0.933					
E-1	LL	7	0	1	0.934	30	0.833	0.837	1.191
	LW	8	2	0.786					
	LVD	7	0	1					
	PL	7	0	1					
	PD	8	4	0.786					
	TLL	7	0	1					
	LM	7	0	1					
	LSI	2	0	1					
	LA	7	0	1					
E-2	LL	8	0	1	1	36	1	0.761	0.989
	LW	8	0	1					
	LVD	8	0	1					
	PL	8	0	1					
	PD	8	0	1					

	TLL	8	0	1					
	LM	8	0	1					
	LSI	8	17	1					
	LA	8	0	1					
E-3	LL	8	0	0.821	0.922	31	0.861	0.903	1.451
	LW	7	0	1					
	LVD	7	0	1					
	PL	7	0	1					
	PD	7	0	1					
	TLL	8	0	0.821					
	LM	7	0	1					
	LSI	3	0	1					
	LA	8	5	0.821					
Understory	LL	8	0	0.929	0.947	34	0.944	0.553	0.803
	LW	6	0	1					
	LVD	8	5	0.929					
	PL	7	2	1					
	PD	8	0	0.929					
	TLL	8	0	0.929					
	LM	8	0	0.929					
	LSI	7	1	1					
	LA	8	0	0.929					
Exposed land	LL	8	0	0.929	0.947	34	0.944	0.830	1.310
	LW	8	0	0.929					
	LVD	7	0	1					
	PL	7	0	1					
	PD	8	2	0.929					
	TLL	8	0	0.929					
	LM	8	0	0.929					
	LSI	6	0	1					
	LA	8	0	0.929					

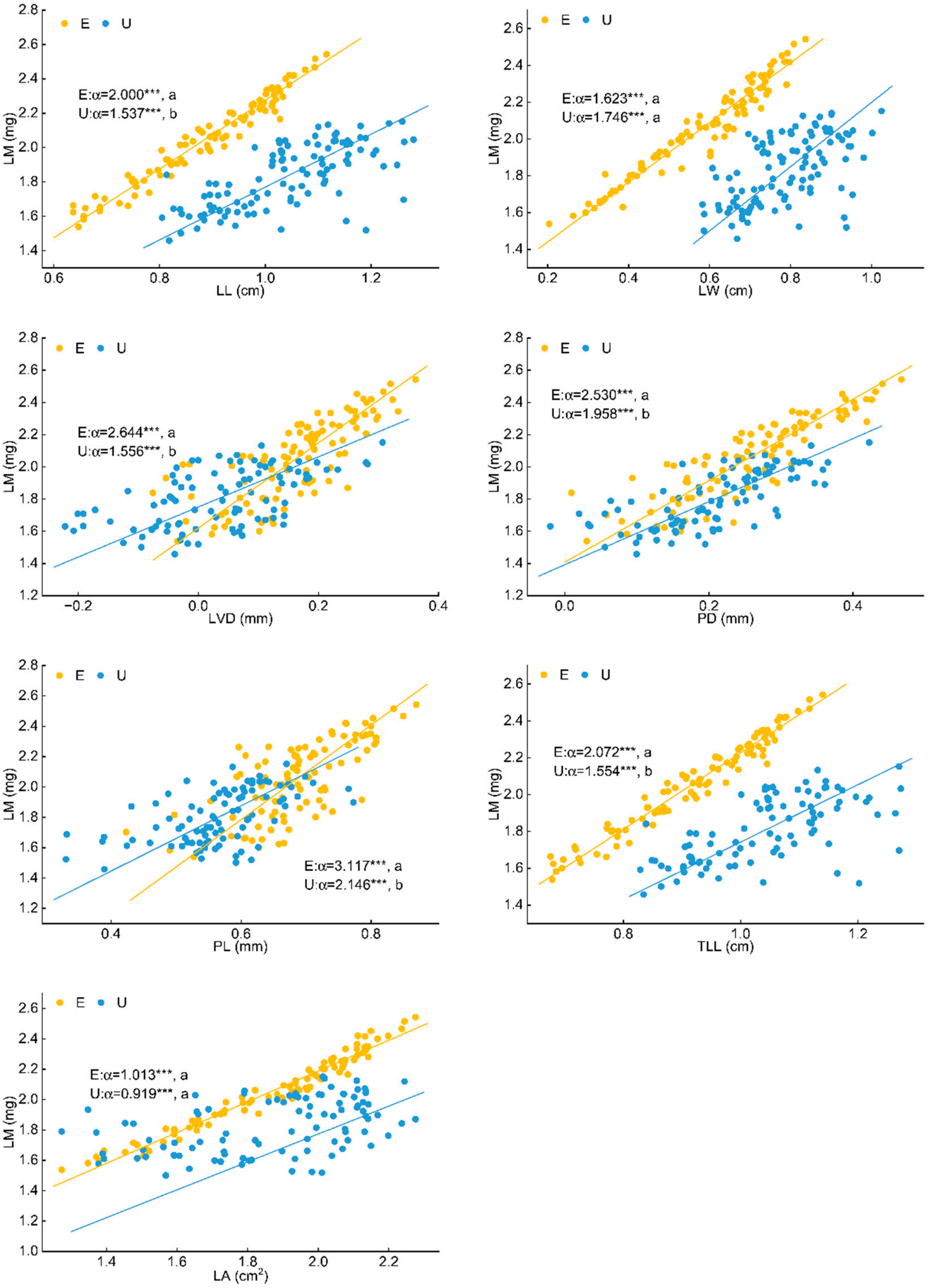

Figure S1. Allometric scaling exponents among leaf mass and main leaf traits (log_{10}-transformed) of *Crotalaria spectabilis* in different habitats. U: understory habitat, E: exposed land habitat. Letters denote significant differences (**P* < 0.05, ***P* < 0.01, ****P* < 0.001).

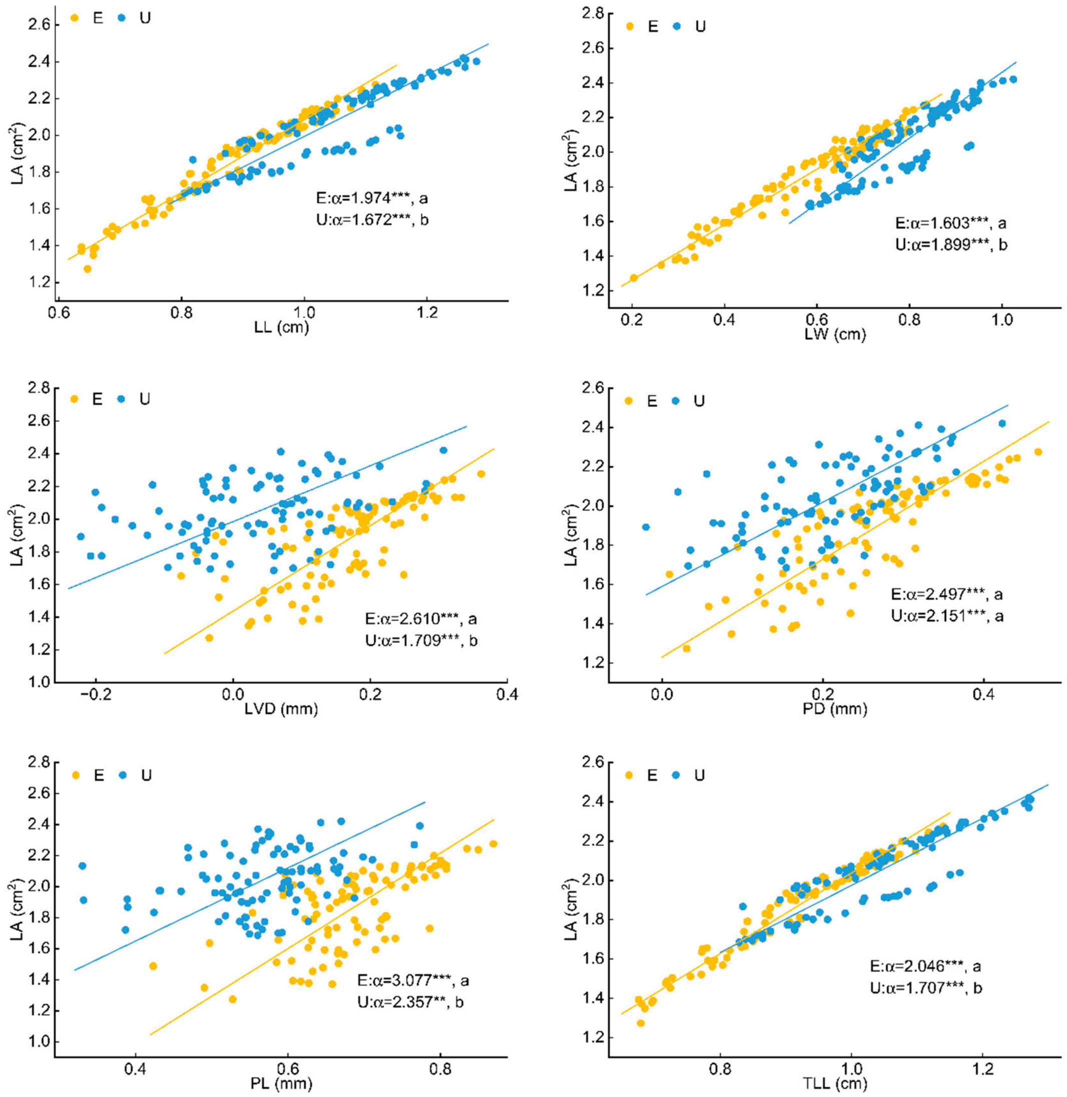

Figure S2. Allometric scaling exponents among leaf area and main leaf traits (log_{10}-transformed) of *Crotalaria spectabilis* in different habitats. U: understory habitat, E: exposed land habitat. Letters denote significant differences (*P < 0.05, **P < 0.01, ***P < 0.001).

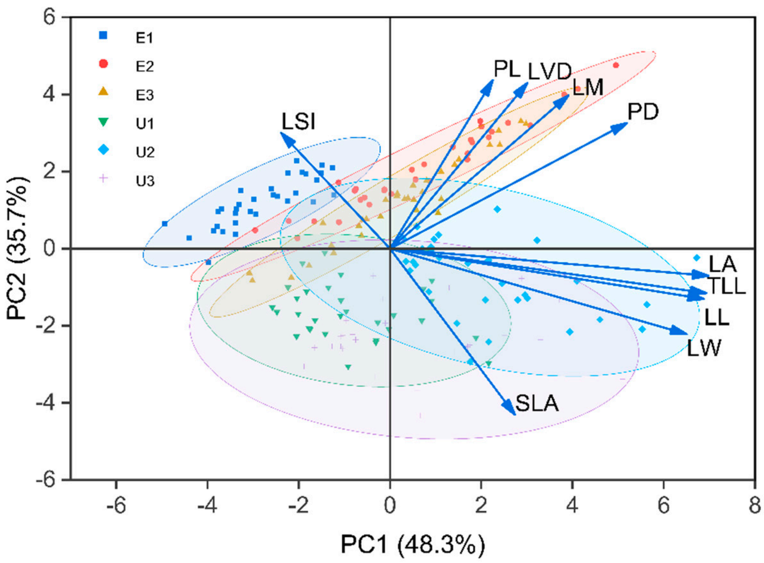

Figure S3. Principal Component Analysis indicating overall difference in leaf traits of *Crotalaria spectabilis* in two habitats and three canopy positions based on trait matrix. U1 (upper canopy), U2 (middle canopy), and U3 (lower canopy) for the understory, and E1 (upper canopy), E2 (middle canopy), and E3 (lower canopy) for exposed land.
